# Non-invasive pulmonary artery pressure estimation by electrical impedance tomography in a controlled hypoxemia study in healthy subjects

**DOI:** 10.1038/s41598-020-78535-4

**Published:** 2020-12-08

**Authors:** Martin Proença, Fabian Braun, Mathieu Lemay, Josep Solà, Andy Adler, Thomas Riedel, Franz H. Messerli, Jean-Philippe Thiran, Stefano F. Rimoldi, Emrush Rexhaj

**Affiliations:** 1 Systems Division, Swiss Center for Electronics and Microtechnology (CSEM), Neuchâtel, Switzerland; 2 Signal Processing Laboratory (LTS5), Ecole Polytechnique Fédérale de Lausanne (EPFL), Lausanne, Switzerland; 3 Systems and Computer Engineering, Carleton University, Ottawa, Canada; 4 Department of Paediatrics, Cantonal Hospital Graubuenden, Chur, Switzerland; 5 Department of Paediatrics, Inselspital Bern, University Children’s Hospital, Bern, Switzerland; 6 Department of Cardiology and Clinical Research, Inselspital Bern, University Hospital, Bern, Switzerland; 7 Department of Radiology, University Hospital Center (CHUV) and University of Lausanne (UNIL), Lausanne, Switzerland

**Keywords:** Biomedical engineering, Hypoxia, Cardiovascular diseases, Hypertension

## Abstract

Pulmonary hypertension is a hemodynamic disorder defined by an abnormal elevation of pulmonary artery pressure (PAP). Current options for measuring PAP are limited in clinical practice. The aim of this study was to evaluate if electrical impedance tomography (EIT), a radiation-free and non-invasive monitoring technique, can be used for the continuous, unsupervised and safe monitoring of PAP. In 30 healthy volunteers we induced gradual increases in systolic PAP (SPAP) by exposure to normobaric hypoxemia. At various stages of the protocol, the SPAP of the subjects was estimated by transthoracic echocardiography. In parallel, in the pulmonary vasculature, pulse wave velocity was estimated by EIT and calibrated to pressure units. Within-cohort agreement between both methods on SPAP estimation was assessed through Bland–Altman analysis and at subject level, with Pearson’s correlation coefficient. There was good agreement between the two methods (inter-method difference not significant (*P* > 0.05), bias ± standard deviation of − 0.1 ± 4.5 mmHg) independently of the degree of PAP, from baseline oxygen saturation levels to profound hypoxemia. At subject level, the median per-subject agreement was 0.7 ± 3.8 mmHg and Pearson’s correlation coefficient 0.87 (*P* < 0.05). Our results demonstrate the feasibility of accurately assessing changes in SPAP by EIT in healthy volunteers. If confirmed in a patient population, the non-invasive and unsupervised day-to-day monitoring of SPAP could facilitate the clinical management of patients with pulmonary hypertension.

## Introduction

Pulmonary hypertension (PH) is a pathophysiological condition characterized by a persistent and abnormal elevation of mean pulmonary artery pressure (PAP) above 25 mmHg, as assessed by right heart catheterization^1^. Chronic PH, with left heart disease as its most common cause, affects at least 0.3% of the general population and more than 60% of patients with moderate or severe heart failure^2,3^. The long-term follow-up of patients with PH relies in part on spot PAP measurements performed during clinic visits at intervals of several months^1^. However, assessing changes in PAP on a daily basis has shown to improve outcomes in patients with left heart failure equipped with an implantable monitor^4^, as increases in intracardiac pressures occur days or weeks before the onset of symptoms^5^. The availability of day-to-day trends in PAP for the clinician allows anticipating worsening conditions, thereby significantly reducing hospitalization rates^4^. However, while implantable monitors provide valuable insights in the patient’s response to therapy and allow earlier adaptation of treatment, they remain a highly invasive solution. Transthoracic echocardiography (TTE)—although non-invasive—is incompatible with daily unsupervised monitoring because of its dependency on a trained operator to perform the measurement and its inapplicability in 20–50% of patients due to unmeasurable tricuspid regurgitation^6,7^. A non-invasive solution able to provide PAP estimates on a day-to-day basis without supervision does currently not exist.

In this context, we have recently proposed a non-invasive and unsupervised method for the continuous monitoring of PAP^8^ and demonstrated its feasibility in a model-based simulation^9^. Our technique is based on electrical impedance tomography (EIT), a non-invasive and radiation-free medical imaging method allowing continuous monitoring at the bedside^10^. The main strength of EIT resides in its ability to monitor changes in the intra-thoracic distribution of electrical impedance with a high temporal resolution. Thus, impedance changes induced by respiratory and cardiovascular activity (e.g. changes in air volume in the lungs or in blood volume in the heart and arteries) can be monitored in real-time. In particular, changes in the pulsatility of the pulmonary arteries have shown to produce variations in EIT images in the pulmonary region, allowing assessment of PAP-related information from EIT^11^. We present hereafter the first experimental evidence of our method in a preclinical controlled hypoxemia study in 30 healthy participants.

## Methods

The study was conducted in accordance with the amended Declaration of Helsinki. The protocol was approved by the Ethical Committee of the Canton of Bern (KEK), Switzerland (KEK Application 200/12), and registered at ClinicalTrials.gov (NCT02969486). All participants provided written informed consent.

### Measurement principle

The velocity at which a pressure wave travels along an artery—the pulse wave velocity (PWV)—is linked to the elastic properties of the arterial wall^12^. As the arterial wall stiffens, the PWV increases and a larger blood pressure pulse is required to expand the wall. Thus, PWV and blood pressure are intrinsically linked. The PWV between two locations separated by a distance D along an arterial pathway can be calculated as PWV = D/PTT, where PTT is the pulse transit time. For blood pressure-related applications, estimating D—which is considered constant—is unnecessary as a calibration function *f*(·) is eventually required for converting the estimated PWV values to pressure units (mmHg). Therefore, monitoring changes in PAP using the PWV directly relies on monitoring changes in PTT: PAP = *f*(PTT). In our approach, the first arterial location is the pulmonary valve, and the second one a distal location in the lungs. As further explained hereafter, we use EIT to detect the arrival of the pressure pulse in the lungs. However, detecting in a non-invasive way the time of opening of the pulmonary valve is particularly complex. This is why in practice the PTT is often approximated by the pulse arrival time, which uses the R-wave peak of the electrocardiogram (ECG) as reference time instead of the opening of the valve^13^. In the present study, an ECG signal was thus measured along with the EIT data, and the pulse arrival time was used as a surrogate measure of the PTT.

### Participants and experimental protocol

We enrolled thirty healthy participants in our study. Exclusion criteria were age below eighteen, known heart or pulmonary disease, pacemaker or implantable cardioverter defibrillator, pregnancy or lactation.

In order to induce PAP variations, the subjects breathed nitrogen-enriched air through a mask connected to an altitude simulator (AltiTrainer, SMTEC, Switzerland). Normobaric hypoxemia causes vasoconstriction in the pulmonary arterial tree and therefore induces elevations of PAP. The peripheral capillary oxygen saturation (SpO_2_) of the subjects was continuously monitored using the Radical-7 device (Masimo, USA), whereas their systolic pulmonary artery pressure (SPAP) was measured periodically by TTE. Each subject was lying in supine position and underwent seven 2-min EIT and ECG measurements at various oxygen saturation levels: two measurements in normoxemia (~ 540 m, SpO_2_ ≥ 95%), two measurements in mild hypoxemia (~ 4200 m, 90% > SpO_2_ ≥ 80%), two measurements in profound hypoxemia (~ 5400 m, 80% > SpO_2_ ≥ 65%), and at the end again one baseline measurement in normoxemia. Immediately after each EIT measurement, the subjects were asked to turn on their left side to measure their SPAP by TTE.

The echocardiographic measurements were performed with a real-time, phased-array sector scanner (Acuson Sequoia 512, Siemens, Germany) with an integrated color Doppler system and transducers containing crystal sets for 2-dimensional imaging (5.0 MHz with second harmonic technology) and for continuous-wave Doppler recording (2.5 MHz). After localization of tricuspid regurgitation via Doppler color flow imaging, the maximal tricuspid regurgitation velocity v_max_ was measured by means of continuous-wave Doppler ultrasound. The right atrioventricular pressure gradient ΔP was estimated using the modified Bernoulli equation (ΔP = 4 v_max_^2^), as previously validated by our group in hypoxemia against invasive measurements^14^. The SPAP was estimated as ΔP + RAP, where RAP (right atrial pressure) was assumed to be 3 mmHg^15^. To reduce the impact of TTE’s imprecision^16^, each reported SPAP value is the average of at least three consecutive measurements. The maximal deviation among averaged values was verified to be systematically ≤ 4 mmHg. The EIT data were acquired using the 16-electrode Goe-MF II device (CareFusion, Germany) in combination with 16 gel electrodes (Ambu BR-50-K, Ambu, Denmark) placed around the participants’ thorax in a transversal plane at the level of the fifth intercostal space. An ECG was synchronously recorded using the ECG100C module (Biopac Systems, Inc., USA) and fed into the auxiliary input of the EIT device. The EIT data were acquired using an injection frequency of 100 kHz and a frame rate of 25 per second. The ECG signal was sampled at 325 Hz. The EIT data were reconstructed into images of 64 × 64 pixels with Matlab (MathWorks, Natick, USA) using the EIDORS toolbox^17^. The reconstruction was performed using the 2.5D human thorax model of EIDORS and the GREIT algorithm with the recommended settings^18^.

### Estimation of the pulmonary PTT

**Figure 1:**
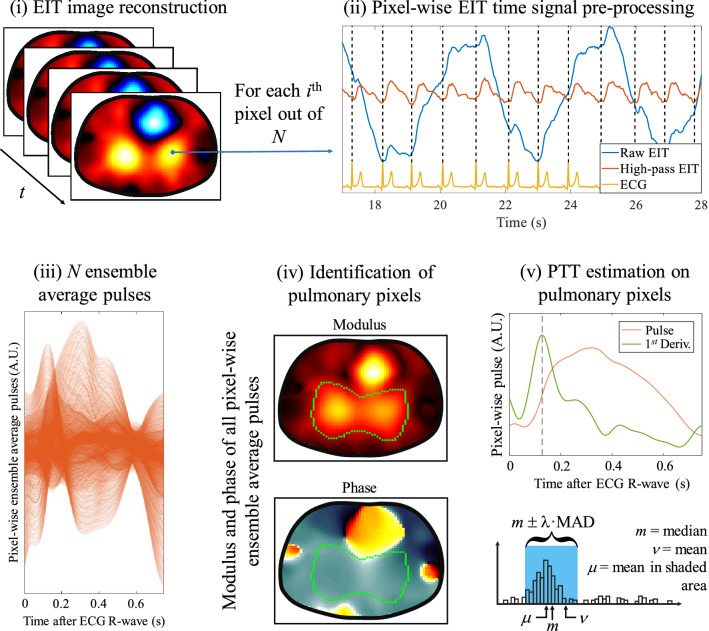
Estimation of the pulmonary PTT (as the pulse arrival time) from ECG and EIT data. After (**i**) EIT image reconstruction, each pixel-wise EIT time signal is (**ii**) high-pass filtered and summarized to one representative pulse through ECG-gated ensemble averaging, resulting in (**iii**) *N* cardio-synchronous ensemble average pulses for the *N* pixels of the EIT image sequence. (**iv**) The modulus and phase of all *N* ensemble average pulses is calculated and a pulmonary region of interest of *M* pixels is obtained by finding all pixels with significant pulsatile amplitude showing coherent phase with the average cardiac-related EIT signal. (**v**) For each of the *M* pulmonary pixels, the PTT is estimated using the maximum of its first time derivative. Finally, the global pulmonary PTT is obtained by averaging the PTT values of all *M* pulmonary pixels after outlier rejection using the MAD method. *ECG* electrocardiography, *EIT* electrical impedance tomography, *PTT* pulse transit time, *MAD* median absolute deviation.

The estimation of the pulmonary PTT for each 2-min EIT recording was fully automated and is illustrated in Fig. 1. As a first step, the EIT time signal at each pixel was high-pass filtered (0.65 Hz) using zero-phase filtering. Each 2-min EIT signal at each pixel was then averaged to one representative pulse through ECG-gated ensemble averaging^19^. Then, a pulmonary region of interest (ROI), defined as those pixels depicting a lung-like behavior and a significant pulsatility, was automatically segmented from the EIT images. To do so, a similar approach to the one proposed previously was implemented^9,20^. The pulmonary ROI was defined as those pixels showing coherent phase with the average cardiac-related EIT signal (average signal over all pixels). The ROI was then limited to pixels showing a pulsatile amplitude exceeding a threshold equal to the first quartile of the distribution of amplitude values. The pulmonary PTT was estimated at each pixel in the ROI from the ensemble average pulse using the maximum of its first time derivative. Possible outliers were automatically rejected using the median absolute deviation method^21^. The final pulmonary PTT was obtained by averaging all non-rejected PTT estimates.

### EIT-based SPAP estimation

In order to translate our PTT estimates into actual SPAP values, we made use of a calibration function *f*(·) linking both quantities as SPAP = *f*(PTT). Although the function *f*(·) is typically an inverse exponential^22,23^, monitoring relatively small PAP changes around a certain baseline value allows to locally approximate *f*(·) by a simple affine function: SPAP = α·PTT + β^24^. In this study we considered the practical use case where only the β coefficient is adjusted for each subject, while the α coefficient remains the same for all subjects. The value of the α coefficient was obtained through linear regression of the PTT values onto their corresponding SPAP values at a cohort level, leading to an α value of − 710 mmHg/s. Being a simple offset, the β coefficient does not require inducing PAP variations to be calibrated and was therefore adjusted for each subject using the baseline measurements in normoxemia only, as β = average (SPAP − α·PTT). We also investigated the calibration scenario where the α coefficient is adjusted at a subject level; although impractical as it requires inducing PAP variations in the patient, this analysis allows quantifying the best achievable agreement using our proposed approach.

### Statistical analysis

The agreement between the estimated EIT-derived SPAP values (SPAP_EIT_) and the TTE-derived SPAP values (SPAP_TTE_) at a cohort-wise level was evaluated via Bland–Altman analysis^25^. Using SPAP_TTE_ as reference, the accuracy of SPAP_EIT_ was evaluated using the statistical bias (average value of the differences between both methods), and its precision was evaluated using the standard deviation (SD) of the differences. These accuracy and precision assessments were also performed after grouping all measurements into three groups (normoxemia, mild hypoxemia, and profound hypoxemia). Paired-sample *t*-tests were used to determine if the difference between both methods was significant (at the 5% significance level). The agreement of SPAP_EIT_ with SPAP_TTE_ was also evaluated at a subject-wise level. Finally, the ability of EIT in tracking SPAP changes in the individual participant was evaluated using Pearson’s correlation coefficient (5% significance level). The distribution of the subject-wise performance values was reported using the median, the first (*Q*_1_) and third (*Q*_3_) quartiles, and the range (minimal and maximal values).

## Results

Three volunteers had no measurable tricuspid regurgitation—needed for the estimation of SPAP_TTE_—and in three subjects the EIT signals were non-interpretable for technical reasons, leaving us with a total of 24 subjects. In two participants, the recording of the ECG signal during the first measurement in normoxemia failed, leaving us with 6 measurements instead of 7 for these two subjects. Thus, a total of 166 paired TTE-EIT data points were obtained (70 in normoxemia, 48 in mild hypoxemia, and 48 in profound hypoxemia). SPAP_TTE_ was significantly increased (*P* < 0.001) during mild (25.1 ± 4.3 mmHg) and profound hypoxemia (32.0 ± 5.2 mmHg) as compared to baseline normoxemic values (19.5 ± 2.9 mmHg). The characteristics of the study participants are presented in Table 1. None of the participants suffered from PH, nor any complication during the tests. Systemic blood pressure and cardiac function were within the normal range in all participants.

**Table 1:** Biometric characteristics of the 24 participants.

Subject characteristics (n = 24)	Mean (SD), or count (percentage)
Age (year)	33.6 (9.1)
Height (cm)	175.4 (5.7)
Weight (kg)	74.3 (11.5)
Body mass index (kg/m^2^)	24.1 (3.1)
Gender, male	20 (83%)
Active smoking	1 (4%)
Systemic arterial pressure (mmHg)	
Systolic	126.2 (14.2)
Diastolic	74.8 (8.8)
Heart rate (bpm)	60.2 (9.8)
Peripheral oxygen saturation, SpO_2_ (%)	
Normoxemia	96.4 (1.2)
Mild hypoxemia	85.4 (4.0)
Profound hypoxemia	74.3 (5.4)
TTE-derived systolic pulmonary artery pressure (mmHg)	
Normoxemia	19.5 (2.9)
Mild hypoxemia	25.1 (4.3)
Profound hypoxemia	32.0 (5.2)

The data are given as “mean (standard deviation, SD)”, or as “count (percentage)” when indicated. For peripheral oxygen saturation (SpO_2_) and systolic pulmonary artery pressure, the data are provided as a function of the saturation level: normoxemia (SpO_2_ ≥ 95%), mild hypoxemia (90% > SpO_2_ ≥ 80%), and profound hypoxemia (80% > SpO_2_ ≥ 65%).

*TTE* transthoracic echocardiography.

**Figure 2:**
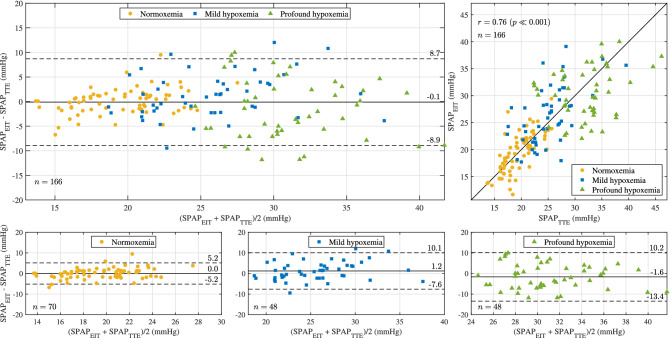
Upper left: Bland–Altman plot depicting the cohort-wise agreement between the EIT-derived SPAP (SPAP_EIT_) and the TTE-derived SPAP (SPAP_TTE_). Each type of symbol represents an oxygen saturation level (SpO_2_), showing n = 70 paired values in normoxemia (filled circle, SpO_2_ ≥ 95%), n = 48 paired values in mild hypoxemia (filled square, 90% > SpO_2_ ≥ 80%), and n = 48 paired values in profound hypoxemia (filled triangle, 80% > SpO_2_ ≥ 65%), for a total of n = 166 paired values. In terms of accuracy, an average difference (bias) of − 0.1 mmHg was found between both methods and is depicted as a solid line. In terms of precision, the standard deviation (SD) of the paired differences was found to be 4.5 mmHg, resulting in 95% limits of agreement (bias ± 1.96 SD) of [− 8.9, 8.7] mmHg, depicted as dashed lines. Upper right: Correlation plot between SPAP_EIT_ and SPAP_TTE_, with a cohort-wise Pearson’s correlation coefficient of 0.76 (P $\ll$ 0.001). Bottom: Bland–Altman plots for each SpO_2_ level separately. *EIT* electrical impedance tomography, *TTE* transthoracic echocardiography, *SPAP* systolic pulmonary artery pressure.

Figure 2 (upper left) depicts the Bland–Altman plot for the entire cohort. Each SpO_2_ level (normoxemia, mild hypoxemia, and profound hypoxemia) is represented by a different symbol. The solid line shows the cohort-wise accuracy (bias) of − 0.1 mmHg, and the two dashed lines encompass the 95% limits of agreement (bias ± 1.96 SD), thus depicting a cohort-wise precision (SD) of 4.5 mmHg. Bland–Altman plots for each SpO_2_ level are shown in the lower panel of Fig. 2. Finally, the upper right panel depicts the correlation plot between SPAP_EIT_ and SPAP_TTE_, with a cohort-wise Pearson’s correlation coefficient of 0.76 (P $\ll$ 0.001).

Table 2 reports the cohort-wise agreement between SPAP_EIT_ and SPAP_TTE_ at various levels of oxygen saturation. In all cases, the null hypothesis that the paired differences between both methods come from a normal distribution with mean equal to zero could not be rejected at the 5% significance level.

**Table 2:** Agreement between the EIT-derived and TTE-derived SPAP estimates at the cohort-wise level.

Cohort-wise measurements	TTE (mmHg)	EIT (mmHg)	EIT − TTE (mmHg)
All measurements combined (n = 166)	24.7 ± 6.6	24.6 ± 6.4	− 0.1 ± 4.5^†^
Normoxemia (n = 70)	19.5 ± 2.9	19.5 ± 3.7	0.0 ± 2.6^†^
Mild hypoxemia (n = 48)	25.1 ± 4.3	26.3 ± 5.4	1.2 ± 4.5^†^
Profound hypoxemia (n = 48)	32.0 ± 5.2	30.4 ± 4.7	− 1.6 ± 6.0^†^

The distributions over the entire cohort (n = 166 paired measurements) are shown as “mean ± standard deviation”. The agreement is also depicted as a function of the oxygen saturation level (SpO_2_), comparing the distributions in normoxemia (SpO_2_ ≥ 95%), mild hypoxemia (90% > SpO_2_ ≥ 80%), and profound hypoxemia (80% > SpO_2_ ≥ 65%). In all cases, the distribution of the differences (EIT − TTE) between both methods showed to be non-significantly different from a zero-mean normal distribution at the 5% significance level.

*EIT* electrical impedance tomography, *TTE* transthoracic echocardiography, *SPAP* systolic pulmonary artery pressure.

^**†**^Paired-sample *t*-test: Not significant at the 5% significance level (*P* > 0.05).

Table 3 shows the agreement and correlation between SPAP_EIT_ and SPAP_TTE_ for all participants at the subject level. The distributions of all values are depicted using the three quartiles (*Q*_1_, median, and *Q*_3_), and the range (minimal and maximal values). Thus, a median per-subject difference of 0.7 ± 3.8 mmHg (bias ± SD) was found between both methods, and a median Pearson’s correlation coefficient of 0.87. The correlation was found to be significant at the 5% significance level for all participants but four, three of which with *P* < 0.10 and one, with the lowest per-subject correlation of 0.63, with *P* = 0.12.

**Table 3:** Agreement between the EIT-derived and TTE-derived systolic pulmonary artery pressure estimates at the subject-wise level.

Subject-wise measurements	Minimum	*Q*_1_	Median	*Q*_3_	Maximum
Mean of the differences (mmHg)	− 5.4	− 2.6	0.7	1.4	4.8
SD of the differences (mmHg)	1.6	2.9	3.8	4.7	6.1
Pearson’s correlation coefficient	0.63	0.78*	0.87*	0.92*	0.99*

For each subject, the mean and standard deviation (SD) of the paired differences between both methods was computed, along with Pearson’s correlation coefficient. The distributions over all participants are shown using the three quartiles (*Q*_1_, median, and *Q*_3_), and the range (minimal and maximal values).

*EIT* electrical impedance tomography, *TTE* transthoracic echocardiography.

*Significant at the 5% significance level (*P* < 0.05).

In order to evaluate the best achievable agreement between SPAP_EIT_ and SPAP_TTE_, the calibration scenario where the α coefficient is adjusted at a subject level was also investigated. The cohort-wise accuracy (bias) and precision (SD) decreased to 0.0 and 2.8 mmHg, respectively, and the cohort-wise Pearson’s correlation coefficient increased to 0.90 (P $\ll$ 0.001). At a subject level, a median per-subject difference of 0.0 ± 2.6 mmHg (bias ± SD) was found between SPAP_EIT_ and SPAP_TTE_. The subject-wise Pearson’s correlation coefficients being unaffected by the value of the α coefficient, remained unchanged.

## Discussion

There is currently no non-invasive and unsupervised solution for the day-to-day monitoring of pulmonary artery pressure in patients with chronic pulmonary hypertension. This study presents experimental evidence of the feasibility to monitor SPAP non-invasively using EIT in 24 healthy subjects through the measurement of the pulmonary PTT. Changes in SPAP were induced by hypoxemic vasoconstriction and compared to SPAP values obtained from echocardiographic measurements. Results demonstrate no significant difference between both methods, irrespective of the level of SPAP, from normoxemia to profound hypoxemia. This agreement was also verified at a subject level, along with strong and significant subject-wise Pearson’s correlation coefficients, thereby confirming the ability of our proposed approach in tracking intra-subject changes in PAP via EIT. When adjusting the α calibration coefficient at a subject level, a general ~ 35% precision improvement was observed; however, given the impractical applicability of such a calibration procedure, these results only serve to illustrate the best achievable agreement obtainable with our method.

While our results demonstrate excellent accuracy (bias of − 0.1 mmHg), their moderate precision (standard deviation of 4.5 mmHg) may lead to occasional large differences in individual measurements. A part of this imprecision may be attributed to our use of TTE as reference^16^, highlighting the need for further comparison studies against an invasive reference. However, because EIT measures continuously and without supervision, its precision can easily be improved by repeating and averaging measurements, as averaging $N$ measurements decreases the standard deviation of the error by a factor $\sqrt{N}$^26^. For instance, averaging 3 measurements already brings the standard deviation down from 4.5 to 2.6 mmHg. Considering 2-min measurements as is the case in our study, this suggests that wearing an EIT system for only a few minutes is sufficient to obtain averages with higher precision.

Cardiovascular applications of EIT have received increasing attention over the last decade, from investigations on cardiac output to pulmonary perfusion^27–30^. The use of EIT for monitoring patients with PH has been proposed previously by Smit et al*.*^11^. However, although they, with high accuracy, could discriminate between patients and healthy subjects, no correlation was found between EIT-based features and the severity of the disease. Furthermore, as all their patients suffered from advanced disease, they could not extrapolate their results to early stages. In contrast, our PWV-based approach for PAP monitoring is thought to be particularly well-suited for detecting early changes in pulmonary hemodynamics. This is due to the direct link between PWV and arterial distensibility^12^, which is an even stronger predictor of survival than PAP itself^31,32^. In addition, the inverse exponential nature of the PTT-PAP relationship suggests higher measurement sensitivity at lower pressures, from mild to moderate PH. Conversely, our approach may suffer from lower sensitivity at severe hypertensive stages. From a model-based study^9^, this decrease in the sensitivity of our approach may begin at mean PAP values of ~ 40–50 mmHg (SPAP ~ 60–75 mmHg), which we did not investigate in the present study. This is concordant with findings from an intravascular ultrasound study^23^, where arterial distensibility compared to PAP progressively reached a plateau starting from a mean PAP of ~ 40 mmHg, as the arteries were approaching their elastic limit. A direct consequence of the non-linear nature of the PTT-PAP relationship is an expectable decrease in the magnitude of the α calibration coefficient as PAP increases, suggesting that applying our proposed approach to a large patient population might require prior training of the α calibration coefficient over a large range of PAP values covering the physiological range.

When compared to the only currently available non-invasive PAP measuring solution that is TTE, EIT has several advantages, such as its lower cost, and its ability to provide unsupervised monitoring. While TTE requires an experienced echocardiographer for accurate measurement of SPAP, no particular training other than attaching an electrode belt to a patient is required for EIT. Furthermore, the applicability of EIT exceeds that of TTE, for which 20–50% of patients have no measurable tricuspid regurgitation^6,7^. Lastly, because EIT provides continuous measurements, our method has the potential of detecting minute variations in pulmonary hemodynamics, otherwise missed by intermittent methods such as TTE. This could prove particularly useful in contexts of care such as intensive care units, where the use of right heart catheterization has been sharply reduced over the last decades after several randomized controlled trials failed to demonstrate any decrease in morbidity and mortality following its use in critically ill and hemodynamically unstable patients^33–36^. In this context, EIT could be used to carry on the monitoring of PAP after earlier removal of the catheter, thereby reducing the risks associated with its maintenance. In patients lacking invasive monitoring, EIT could provide valuable information by measuring continuous PAP trends.

A potentially limiting aspect of our approach is that it does not directly estimate absolute values of PAP, but absolute changes of PAP around an arbitrary baseline value. Setting this baseline value requires a calibration, such as a TTE-derived measurement of the patient’s SPAP during a visit in the clinics. In other contexts such as the aforementioned intensive care settings, the EIT device could simply be calibrated using the invasive PAP before catheter removal.

Another potential limitation of our approach is the use of the pulse arrival time as a surrogate of the PTT, which may make it sensitive to factors affecting the pre-ejection period, such as ventricular contractility changes^13^. Also, EIT belt position changes (e.g. rotations or vertical shifts) may affect the accuracy of our approach, mainly at severe hypertensive levels^37^. The use of 3D EIT^38^, shirt-embedded EIT belts^39^, or torso shape detection methods^40^ are various possible strategies that should help minimize the influence of such belt displacements. The well-known limited signal-to-noise ratio of cardiovascular-related EIT signals^10^ is another aspect that may occasionally challenge our approach, as observed in the three subjects rejected for this reason prior to analysis in our study (10% failure rate). Also, considering one single global PTT value in our approach for the entire lung vasculature may lead to inaccuracies in cases of heterogeneous PTT distributions, e.g. in case of ventilation/perfusion mismatch. In such cases, an analysis based on subregions of interest should be performed, but is out of the scope of the present study, where healthy subjects were investigated. Lastly, the performance of our approach needs to be assessed versus the invasive gold standard, and on a patient population, for a formal validation. Preliminary results compared to the invasive method proved promising in neonatal lambs^41^.

In conclusion, the results of our study suggest that changes in PAP may be reliably monitored by a novel, non-invasive, continuous and unsupervised approach based on EIT. If confirmed in a patient population, these findings may open new perspectives for the day-to-day follow-up of patients with pulmonary hypertension, offering a better control of early disease progression and symptoms. Our method may also be particularly useful in intensive care settings, where the benefit-to-risk ratio associated with an invasive monitoring approach often dissuade clinicians.
